# Application of contrast-enhanced ultrasound in minimally invasive ablation of benign thyroid nodules

**DOI:** 10.1016/j.jimed.2021.12.008

**Published:** 2022-02-26

**Authors:** Jiaying Cao, Peili Fan, Feihang Wang, Shuainan Shi, Lingxiao Liu, Zhiping Yan, Yi Dong, Wenping Wang

**Affiliations:** a Department of Ultrasound, Zhongshan Hospital, Fudan University, Shanghai, China; b Department of Interventional Radiology, Zhongshan Hospital, Fudan University, Shanghai, China; c Shanghai Institute of Medical Imaging, Shanghai, China

**Keywords:** Ultrasound, Contrast agent, Thyroid, Microwave ablation, Radiofrequency ablation

## Abstract

**Objective:**

This study aimed to investigate the application value of contrast-enhanced ultrasound (CEUS) before and after minimally invasive ablation procedures for benign thyroid nodule(s) (BTN).

**Methods:**

This prospective study included patients with BTNs scheduled to undergo ultrasound-guided minimally invasive ablation treatment. CEUS was performed before and after ablation (at 1 day, and 1, 6, and 12 months after ablation). Changes in microvascular perfusion and the volume of BTNs were noted and assessed.

**Results:**

Sixty-two patients (62 BTNs), who underwent ablation procedures between June 2016 and August 2020, were included. All lesions were confirmed by biopsy, and histopathological results were obtained before ablation treatment. On preoperative CEUS, the lesions exhibited hyperenhancement (53.23%) or iso-enhancement (46.77%) during the arterial phase, and all lesions exhibited iso-enhancement in the venous and late phases. One day after ablation, none of the BTNs exhibited obvious enhancement on CEUS. One (1.61%) lesion was re-treated due to a nodule-like enhancement area detected by CEUS at the 6-month follow-up. The mean nodular volume reduction rate (VRR) at 1, 6, and 12 months follow-up demonstrated no significant difference between the two ablation groups (microwave ablation versus radiofrequency ablation). Twelve months after ablation, the mean (±SD) VRR of all BTNs was 60.3 ​± ​10.3%.

**Conclusion:**

CEUS helped guide treatment decisions for BTNs before ablation treatment. Moreover, it could also be used to accurately and noninvasively evaluate treatment efficacy.

## 1 Introduction

With the rapid advances and development of medical imaging technologies, the detection rate of thyroid nodules has increased in recent years. According to current guidelines, most thyroid nodules are benign but require regular follow-up.^1^^,^^2^ As a benign thyroid nodule(s) (BTN) grows, it compresses the surrounding trachea, esophagus, and other important organs of the neck. Severe cases can lead to dyspnea, which must be treated in a timely manner.

According to various guidelines, thermal ablation techniques, such as microwave ablation (MWA) and radiofrequency ablation (RFA), are safe and effective minimally invasive treatments for large BTNs.^3^, ^4^, ^5^, ^6^ They have the advantages of minimal invasiveness, rapid recovery, and good cosmetic outcomes.

During thermal ablation of BTNs, real-time ultrasound guidance can accurately locate thyroid nodules to ensure complete ablation. Several studies have reported a significant decrease in nodule volume after ablation.^7^, ^8^, ^9^, ^10^, ^11^, ^12^ However, previous volume comparisons were, in large part, based on conventional ultrasound measurements, which could not sensitively recognize the vital area from the ablated nodule, even when combined with color Doppler imaging. Contrast-enhanced ultrasound (CEUS) can sensitively depict microcirculatory perfusion in the parenchyma organ via injection of a contrast agent. With the aid of CEUS before treatment, it is possible to characterize the blood supply of BTNs and their relationship with the surrounding large vessels.^13^ Changes in blood supply are helpful in judging whether the nodule is completely ablated after treatment. The purpose of the present study was to explore the utility of CEUS before and after minimally invasive ablation treatment of BTNs.

## 2 Methods

This prospective study was approved by the institutional review board of our hospital (approval number: B2020-425). Informed written consent was obtained from all patients before treatment.

### 2.1 Patients

All enrolled patients fulfilled the following inclusion criteria: confirmation of benign nodule status on two separate fine-needle aspiration or core-needle biopsies; no suspicious malignant features on conventional ultrasound examination; concerning nodules growing rapidly or malignant transformation; and refusal to undergo surgery for compression symptoms. Individuals with nodules suspicious for malignancy according to either biopsy or ultrasound examination, those with retrosternal growth, inability to cooperate with complete ablation treatment or CEUS examination, contraindications to CEUS examination, and those with severe risk for bleeding were excluded.

### 2.2 Pre-ablation assessment

Conventional ultrasound and CEUS examinations before and after ablation, as well as during follow-up, were performed using Acuson Sequoia 512 or OXANA2 (Siemens Healthineers, Erlangen, Germany) equipped with a 10L4 or 9L4 linear array transducer, an RS80A (Samsung, Korea) equipped with a LA2-9A linear array transducer, or a Resona 7S (Mindray, Shenzhen, China) equipped with a L14-5WU linear array transducer. Before treatment, each nodule was examined using conventional ultrasound to evaluate volume, location, component (mainly solid, > 50% solid; mainly cystic, > 50% liquid), margin, shape, calcification, and vascularity. The distance from the nodule to the thyroid capsule was also determined.

A bolus dose (2.0 ​mL) of contrast agent (SonoVue, Bracco, Italy) was injected through the middle cubital vein, followed by injection of 5.0 ​mL normal saline. During CEUS, the following features were observed: enhancement intensity (hyperenhancement, iso-enhancement, or hypo-enhancement); enhancement mode (complete or ring enhancement) of the nodule in the arterial (0–30 ​s), venous (31–120 ​s), and late (>120 ​s) phases compared with the surrounding thyroid parenchyma, cystic area, or hemorrhagic area (inside), and hyperenhanced ring around the nodule. If a hyper-enhanced ring was present, the ablation area was enlarged.

### 2.3 MWA procedure

All MWA procedures were performed by an experienced interventional physician with >10 years’ experience with MWA and RFA. An MTC-3C microwave ablation instrument (Viking Medical, Shanghai, China) and MTV-3CAII37 microwave needles were used. The microwave antenna was 16 ​G. Patients were positioned supine with a mildly hyperextended neck on an operating table. Local anesthesia using 1% lidocaine was administered to the surrounding thyroid capsule under ultrasound guidance. If the distance between the tumor and critical cervical structures (trachea, cervical artery, jugular vein, esophagus, and recurrent laryngeal nerve) was <5 ​mm, the hydrodissection technique was used. Fluid aspiration was performed first if there were more cystic components inside the nodule. The output power for MWA was set between 25 ​W and 35 ​W. The “moving-shot technique” was applied during the procedure because most of the BTNs were large, and each nodule was treated by moving the antenna or electrode in a unit-by-unit manner, known as conceptual ablation units.^14^^,^^15^ The water-cooling feature of the system prevents the temperature from rising too quickly to avoid completely ablating the peripheral area of the BTN. Intraoperative complications, including hemorrhage, skin burns, and pain, were recorded.

### 2.4 RFA procedure

RFA was performed using the Medsphere RF Generator S-500 (Medsphere, Shanghai, China) equipped with a 19 ​G electrode. All RFA procedures were performed by the same physician (L.LX) who performed MWA treatment. The RFA equipment and operative procedures were similar to those used for MWA. The RFA electrode must be inserted via the isthmus.

### 2.5 Post-ablation assessment

CEUS was performed 1 day after ablation to evaluate the immediate effectiveness of ablation treatment. To measure the volume and volume reduction rate (VRR), CEUS was performed in the same manner as before and 1, 6, and 12 months after treatment. VRRs were calculated according to the following equation:

VRR = (baseline volume – follow-up volume) / baseline volume ​× ​100%

### 2.6 Statistical analysis

Statistical analyses were performed using SPSS version 20.0 (IBM Corporation, Armonk, NY, USA); differences with *P* ​< ​0.05 were considered to be statistically significant. Categorical data are expressed as percentages and frequencies, and quantitative data are expressed as mean ​± ​standard deviation (SD). The Mann–Whitney test was used to compare quantitative variables and the chi-squared test to compare categorical variables.

## 3 Results

From January 2016 to August 2020, 86 patients with BTNs underwent thermal ablation (RFA or WMA) at the authors’ hospital. Among these, 24 patients with a follow-up time <12 months were excluded. Ultimately, 62 patients with 62 BTNs were enrolled in the study. Conventional ultrasound and CEUS were performed before and after ablation treatment (1 day, and 1, 6, and 12 months).

All BTNs were confirmed by pathological examination using needle biopsy before ablation treatment. A total of 62 patients (52 female, 10 male; mean age, 44.27 ​± ​13.86 years) with 62 BTNs, who underwent invasive ablation, were included in this study. The baseline characteristics of patients with BTNs treated with MWA or RFA are compared in Table 1. There was no significant difference in other characteristics, except that lesion volume was larger in the MWA group than in the RFA group. The incidence rate of complications was similar between the RFA and MWA groups. Skin burns and pain were the most common complications in the two ablation groups. Three patients in the MWA group and two in the RFA group reported skin burns and pain. Although not serious, pain was relieved in five patients the day after the operation without oral supplementation of painkillers. Hoarseness was also reported in the MWA (n ​= ​2) and the RFA (n ​= ​1) groups. Voices of these patients, however, returned to normal during follow-up.

**Table 1:** Baseline characteristics comparison of patients with benign thyroid nodules treated with MWA and RFA.

Characteristics	MWA (n ​= ​43)	RFA (n ​= ​19)	*P*
**Age(years)**	45.44 ​± ​14.42	41.63 ​± ​12.47	0.322
**Gender** (**Female/Male**)	39:4	13:6	0.068
**Location** (**Right/Left**)	26/17	11/8	0.849
**Volume** (**mL**)	8.36 ​± ​6.56	4.43 ​± ​2.23	0.014
**Component** (**mainly solid/mainly cystic**)	22/21	11/8	0.624
**Vascularity** (**Absent/circular/full**)	2/3/38	1/4/14	0.293
**Contrast pattern** (**ring/complete**)	2/41	1/18	1

MWA: microwave ablation, RFA: radiofrequency ablation.

### 3.1 CEUS features before ablation

All BTNs were observed to be cystic solid masses (n ​= ​17) or hypoechoic solid masses (n ​= ​45) on conventional ultrasound imaging. All lesions exhibited a clear boundary and regular shape, with a mean lesion size of 3.28 ​± ​0.72 ​cm. Forty lesions were located in the right lobe of the thyroid and 22 were located in the left lobe. Coarse calcifications were observed in 15 lesions. Discontinuous circular color flow signals could be detected around BTNs in 29 cases, and short linear color flow signals could be detected inside and around the BTNs in 33 cases on color Doppler flow imaging.

**Fig. 1:**
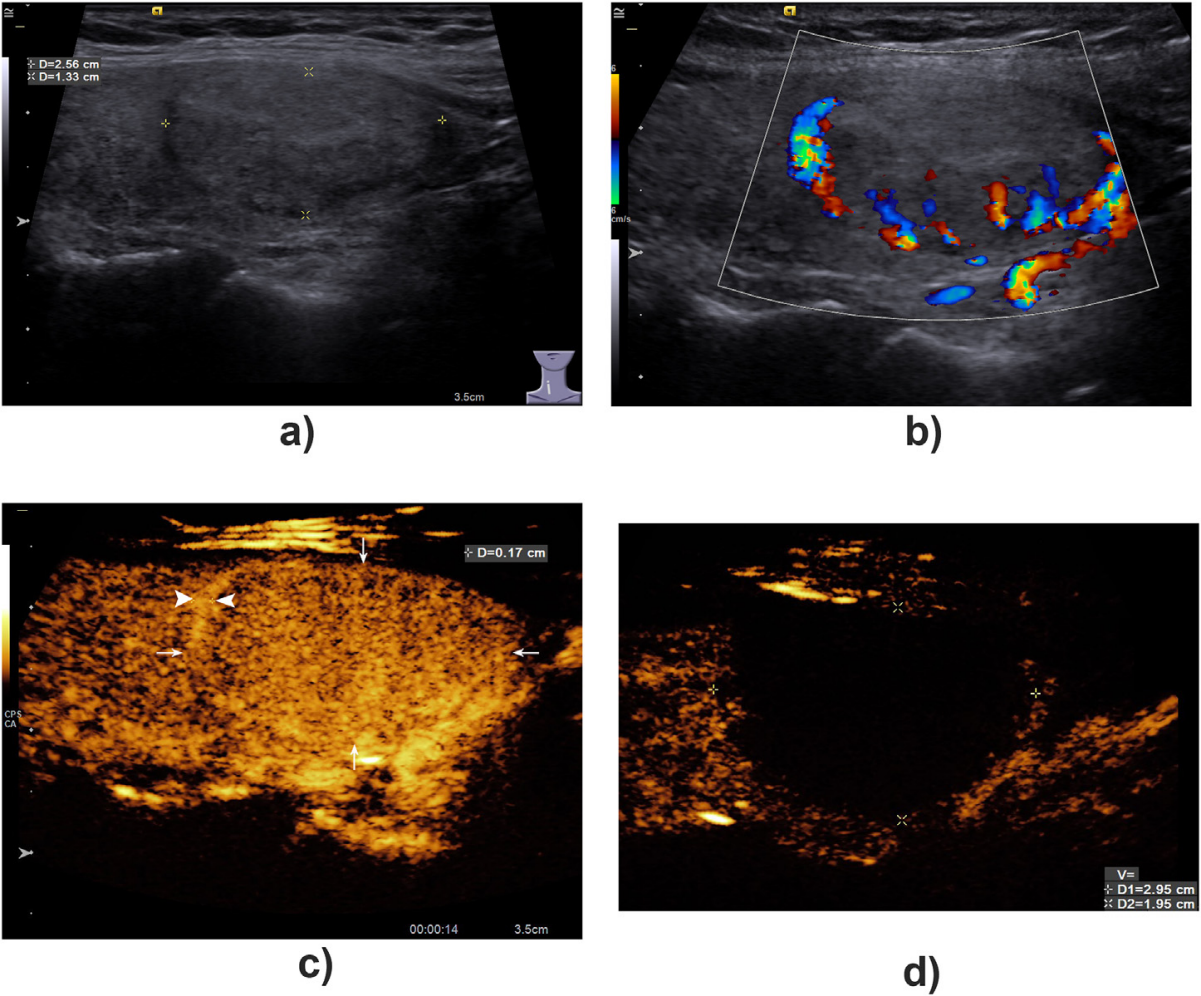
A BTN with a hyper-enhancement ring on CEUS. A 36-year-old woman with a benign nodule in her right lobe of thyroid for 8 years follow-up. The size of the nodule was 2.56 × 1.33 cm before ablation treatment. Conventional ultrasound showed that the nodule was isoechoic with a dark ring around it (A). Linear flow signals were shown around the nodule on color flow image (B). On contrast-enhanced ultrasound, the nodule showed uniform iso-enhancement (arrow), and hyper-enhanced ring (arrowhead) with a thickness of 0.17 cm could be detected around it in arterial phase (C). One month after treatment, CEUS was reexamined. It was found that the non-enhanced area was 2.95 × 1.95 cm and it could completely cover the whole lesion and its surrounding arterial ring (D).

Thirty-three (53.23%) BTNs exhibited typical homogeneous and hyper-enhancement in the arterial phase and iso-enhancement during the venous and late phases; 29 BTNs (46.77%) exhibited synchronous iso-enhancement with surrounding thyroid parenchyma during CEUS. Thirty-six (58.06%) BTNs exhibited a complete hyper-enhancement ring around the lesion in the arterial phase of CEUS, with an average enhancement thickness of approximately 0.17 ​± ​0.06 ​cm (Fig. 1). Conventional ultrasound only revealed anechoic areas in 17 lesions, while CEUS revealed non-enhanced anechoic areas in 23 (37.10%) lesions of all BTNs.

### 3.2 Appearance after ablation

**Fig. 2:**
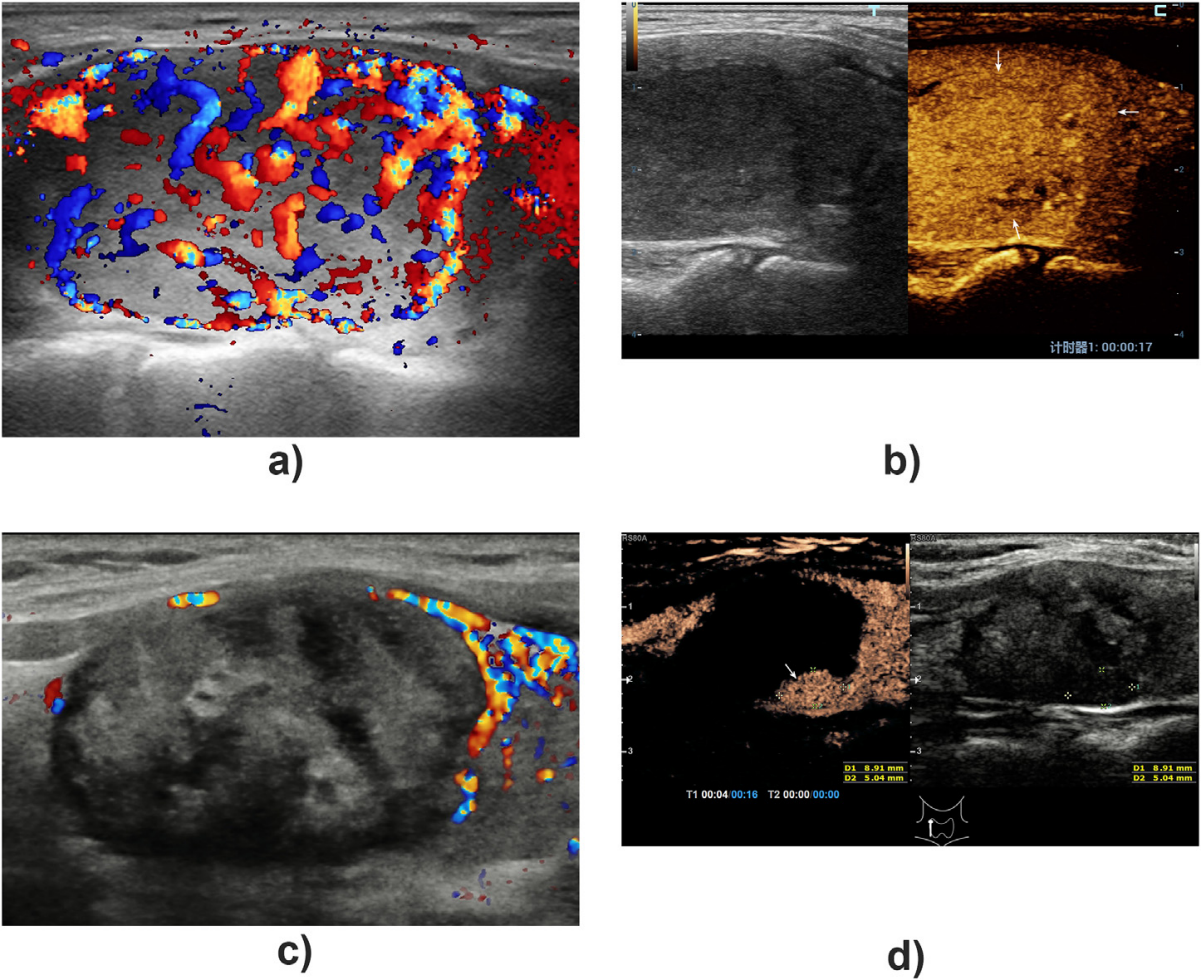
A re-treat BTN. A 55-year-old woman with a benign nodule in her right lobe of thyroid for 12 years follow-up. Abundant flow signals could be detected inside and around the nodule on color flow image (A). On contrast-enhanced ultrasound, the nodule showed uniform iso-enhancement (arrow) in arterial phase before microwave ablation treatment (B)·In follow-up after 6 months, conventional ultrasound reveal a heterogenous hypoechoic nodule without inside color flow signals (C). However, a small nodule-like enhancement area (arrow) was found in the deep position of the ablation area during contrast-enhanced ultrasound (D).

One day after ablation, virtually all BTNs exhibited no obvious enhancement during CEUS. During follow-up, one patient with BTN underwent supplementary ablation. At 1 day and 1 month after treatment, no abnormality was found in the BTN of this patient using CEUS. However, a small nodule-like enhancement area was found in a deep position of the ablated BTN during CEUS at 6 months after treatment of one patient in the MWA group (Fig. 2). After supplementary ablation, the patient was followed up to 1.5 years, and the lesion volume was significantly reduced without enhancement (from 3.92 ​× ​2.46 ​cm to 1.74 ​× ​0.65 ​cm). The mean VRRs in the MWA and RFA groups at 1 day, 1 month, 6 months, and 12 months, respectively, were 0.74 ​± ​2.56% versus (vs.) 1.87 ​± ​4.96% (*P* ​= ​0.340); 24.1 ​± ​11.1% vs. 22.0 ​± ​6.5% (*P* ​= ​0.310); 41.8 ​± ​10.2% vs. 40.3 ​± ​6.6% (*P* ​= ​0.754); and 61.5 ​± ​9.4% vs. 57.7 ​± ​11.9% (*P* ​= ​0.192). The mean VRR of all BTNs was 60.3 ​± ​10.3% at 12 months after ablation.

## 4 Discussion

Thermal ablation technologies use heat to ablate and destroy the target lesion, causing thermal damage to blood vessels in the thyroid, resulting in irreversible coagulation necrosis of the nodule and, finally, reduction of lesion volume.^16^^,^^17^ Jin et al.^18^ concluded that there was no significant difference between the RFA and MWA in terms of operative duration, intraoperative blood loss, hospitalization time, and overall cost. The results of the present study also demonstrated no significant difference between MWA and RFA in treatment effect and patient selection. However, MWA has a wider scope of application, mainly because there are more cystic components in BTNs and their volume is relatively large.

After ablation of BTNs, it is necessary to accurately judge whether there is microcirculatory blood supply in the ablated nodules to evaluate the success of ablation therapy. Complete ablation is important for ensuring the efficacy of ablation treatment for BTNs.^19^ Due to the low sensitivity of conventional gray-scale ultrasound to small blood flow, it is difficult to depict residual blood flow after ablation. Conventional color Doppler ultrasound has an angle dependence on the depiction of color blood flow and cannot completely depict circular artery blood supply around the BTN. Thus, with the advantages of noninvasiveness, operability, high repeatability and no radiation, it is helpful to use CEUS to evaluate microcirculatory blood perfusion in the thyroid tissue to accurately diagnose and differentiate thyroid nodules before surgery,^20^ and to provide important information for the design of clinical follow-up and treatment strategies.

Notably, CEUS played a vital role throughout the study. Preoperative CEUS is helpful in accurately delineating the boundary of thyroid nodules, providing important information for preoperative planning of the appropriate scope of ablation and ensuring the integrity of intraoperative ablation. Because most nodules included in our study were ≥3 ​cm in size, they were characterized by rich vascularization on CEUS, which revealed that most BTNs exhibited hyper-enhancement (53.23%) or iso-enhancement (46.77%) in the arterial phase, and iso-enhancement in the venous phase and delayed phase before ablation treatment. In this study, 36 cases of thyroid adenoma exhibited a complete hyper-enhancement ring around the lesions in the arterial phase. It is important to completely destroy the peripheral arterial ring—if present—during the ablation procedure for BTN. CEUS can dynamically depict the contrast degree and thickness of the arterial ring around the nodule, which can effectively compensate for the limitations of conventional ultrasound. After minimal ablation treatment, it is helpful to use CEUS to simply and accurately depict the size and edge of coagulation necrosis. Results of our study revealed that all BTNs exhibited no obvious enhancement inside the nodule on CEUS, indicating that there was no microvascular perfusion in the nodules, and the lesions were ablated successfully. Results demonstrated that the volume of thyroid nodules decreased at 6 and 12 months after ablation. One day after ablation, CEUS was performed to immediately assess the result of the procedure and to determine whether additional treatment was needed when CEUS revealed zones of residual enhancement. However, it is noteworthy that, due to obvious subcutaneous edema, the location of the lesion was deeper than before treatment, and it is not advisable to apply either conventional ultrasound or CEUS to assess the treatment effect. In our study, hemodynamic changes in BTNs occurred earlier than the change in volume after ablation. At the later follow-up, the actual volume was observed to evaluate the efficacy of thermal ablation. In general, combined with CEUS, RFA and MWA are considered to be effective and safe techniques for treating BTNs.

## 5 Conclusion

Before minimally invasive ablation, CEUS was helpful in the differential diagnosis of thyroid nodules and in determining an appropriate ablation area. After minimally invasive ablation, CEUS was useful for immediate, noninvasive, and sensitive evaluation of the therapeutic effect. Therefore, CEUS is a promising imaging method with potential clinical applications.

## Grant support

Supported by 10.13039/501100001809the National Natural Science Foundation of China (Grant No. 81501471), Clinical Research Plan of SHDC (Grant No. SHDC2020CR1031B, SHDC2020CR4060), and Shanghai Municipal Key Clinical Specialty (Grant No.shslczdzk03501).

## Declaration of competing interest

The authors have no conflicts of interest to declare.
